# [Retracted] Effects of cisplatin on the LSD1-mediated invasion and metastasis of prostate cancer cells

**DOI:** 10.3892/mmr.2023.13027

**Published:** 2023-06-08

**Authors:** Zhi-Yuan Chen, Hui Chen, Tao Qiu, Xiao-Dong Weng, Jia Guo, Lei Wang, Xiu-Heng Liu

Mol Med Rep 14: 2511–2517, 2016; DOI: 10.3892/mmr.2016.5571

Following the publication of this paper, it was drawn to the Editor's attention by a concerned reader that the cell invasion assay data shown for the ‘LSD1siRNA+DDP’ experiment in Fig. 3A on p. 2515 were strikingly similar to data appearing in different form in Fig. 3 in another article written by different authors at different research institutes [Liu Y, Li M, Zhang G and Pang Z: MicroRNA-10b overexpression promotes non-small cell lung cancer cell proliferation and invasion. Eur J Med Res 18: 41, 2013].

Owing to the fact that the contentious data in the above article had already been published prior to its submission to *Molecular Medicine Reports*, the Editor has decided that this paper should be retracted from the Journal. After having been in contact with the authors, they accepted the decision to retract the paper. The Editor apologizes to the readership for any inconvenience caused.

